# Transforming faith-based food pantries in the South Bronx into multiservice health centers

**DOI:** 10.3389/fnut.2026.1913305

**Published:** 2026-09-22

**Authors:** William Suarez-Gomez

**Affiliations:** Department of Health Administration Research and Technology, School of Health Sciences, Health Services & Nursing at Lehman College, City University of New York, New York, NY, United States

**Keywords:** faith-based food pantries, food insecurity, healthcare apartheid, multiservice health centers, public health

## Abstract

Could faith-based food pantries evolve into vital multiservice health centers? In the United States, the South Bronx is the poorest urban congressional district in New York State and experiences significant food insecurity alongside pronounced public health disparities. While food pantries provide nutritional support temporarily, they rarely address the complex social determinants of health that contribute to chronic disease. This paper examines the feasibility, opportunities, and systemic barriers associated with transforming faith-based food operations into integrated, culturally sensitive health hubs. Leveraging their status as trusted community anchors, these organizations could implement a framework that combines clinical healthcare partnerships, co-designed health promotion programs, and diversified funding streams. Despite operational challenges, financial constraints, and institutional mistrust, many faith-based food pantries (FBFP) already extend services beyond food distribution to include spiritual counseling and other forms of support. This proposed structural transformation offers a sustainable, community-based model to address healthcare apartheid, advance health equity, and strengthen integrated community agency.

## Introduction

The domestic emergency-charitable food system—originally conceived as a short-term stopgap to alleviate immediate hunger during acute economic or humanitarian crises (1–3)—has over the last four decades institutionalized into permanent social infrastructure due to systemic gaps in the state welfare framework (4, 5). Driven by this “tertiarization” or outsourcing of social welfare, states increasingly rely on third-sector charitable, non-profit, and faith-based organizations to fulfill basic human rights such as food, health, and dignity (6–9).

Faith based food pantries (FBFP) function as critical, chronic midstream-to-retail “last-mile” food environments that directly influence the dietary quality and health outcomes of millions of low-income families (10–12). This reliance on the third sector mirrors broader domestic structural failures in public welfare delivery systems. For instance, despite maintaining the world's highest per capita healthcare expenditure, the U.S.s remains profoundly fractured: over 26 million residents lack health insurance, approximately 42 million lack adequate access to care, and systemic socioeconomic disparities create localized “healthcare deserts” or “healthcare apartheid” (13–17). Parallel systemic failures manifest where approximately one in eight households with children experiences food insecurity (18, 19). This forces more than 50 million households annually to rely on charitable assistance to supplement basic needs (20–22). Even in New York City—deemed well-positioned to address food distribution vulnerabilities (23)—spending over $500 million annually to serve 200 million meals signals deep, persistent systemic challenges (24).

Nowhere is this intersectional crisis of health and nutritional deprivation more visible than in the South Bronx, New York (25, 26). Home to approximately 728,000 residents—predominantly Hispanic and Black communities—the South Bronx is recognized as the poorest urban congressional district in the state (27). Local food insecurity acutely affects 19% of Bronx residents, with high prevalence rates matching or exceeding this across vulnerable groups, including local CUNY college students (28). Simultaneously, the borough experiences health apartheid, characterized by low-income minorities with disproportionate rates of chronic disorders such as diabetes, obesity, heart disease, asthma, HIV/AIDS, and infant mortality (27, 29–32).

Traditional food pantry systems have historically measured success by the volume of food distributed prioritizing quantity over nutritional quality (22). While several initiatives have proposed frameworks to improve healthy food distribution (33–37), the mere provision of shelf-stable, low/high/fresh-quality items fails to address the multifaceted nature of food insecurity, which is deeply intertwined with broader socioeconomic needs (3, 25). To disrupt this cycle, public health researchers must evaluate whether existing community infrastructure can be repurposed to simultaneously promote health and deliver essential social services (25, 38).

This paper argues that the “tertiarization” of social welfare requires transforming faith-based food pantries (FBFPs) into public health infrastructure. By expanding Section 1115 Medicaid waivers to fund clinical services within FBFPs—and managing this capital through a centralized community-university backend secured by Zero-Knowledge Proof (ZKP) cryptography—this model scales local networks into integrated health hubs without compromising privacy or organizational autonomy. Grounded in a socio-ecological multisystem framework (39) which is rooted in Bronfenbrenner's model, this perspective asserts that multiple systemic layers interact to collectively drive effective program management. Within this context, FBFPs can strategically redistribute capital into community spaces, simultaneously protecting anonymity, preserving religious liberty, and dismantling urban healthcare apartheid.

## Characterizing the charitable food network

### The landscape and vulnerabilities of FBFP

Faith-based organizations represent a substantial share of the social safety net in the U.S., frequently outnumbering their secular counterparts in local social service delivery (9, 40). Participation in these activities is deeply rooted in religious tenets that frame “feeding the hungry” as a fundamental aspect of faith (Table 1) and an expression of communal solidarity (41–43). Consequently, these institutions develop deep roots within their neighborhoods, facilitating the accumulation of social capital and establishing themselves as trusted local landmarks (44). In the Bronx, over 80% of food pantries are operated by faith-based organizations, most of which have been active for over a decade. However, despite their widespread presence, these operations exist within a matrix of environmental, financial, operational, and structural vulnerabilities that limit their systemic impact (45, 46).

**Table 1:** FBFP general comparative variables (landscape).

**Feature**	**Non-denominational**	**Catholic**	**Anglican/episcopal/ baptist**	**Evangelical/pente costals**	**Jewish**	**Muslim**	**Sikh**	**Hindu**	**Buddhist**
Foundational Motivation	Civic duty, human rights, and community welfare.	Social gospel teaching & works of Mercy	Social gospel tradition, stewardship, & community justice	Gospel. The great commission & demonstrating Christ's love	*Tzedakah* (justice/charity) and *Tikkun Olam* (repairing the world)	Obligatory almsgiving (Zakat) and *Sadaqah* (voluntary charity)	*Seva (Selfless service) & Vand Chhako*	*Dāna (Charity) & Karma yoga*.	*Dāna* (generosity) and *Karunā* (compassion to relieve suffering).
Dietary rules	None (adheres to local Std. health codes).	None. (Std. health codes)	None. (Std. health codes)	None. (Std. health codes)	Strictly kosher certified	Strictly halal certified;	Strictly lacto-vegetarian	Strictly vegetarian	Often strictly vegetarian or vegan.
Banned items	N/A	N/A (During Lent)	N/A	Generally, none, but it may vary.	Pork & lard Meat and dairy must be separated.	Pork, lard, and alcohol.	All meat, fish, eggs, alcohol, & tobacco.	All meat, fish, eggs, alcohol (often avoids onions/garlic)	All meat and fish.
Core similarities	Universal service: All of these pantries serve any person in need regardless of their personal religious beliefs. Basic logistics: All rely on a mix of shelf-stable goods, volunteer labor, refrigeration for perishables, and local food bank/pantries supply chains. Dignity focus: All aim to reduce food insecurity and respect the basic human dignity of clients.								
Unique food staples	Standard local grocery surplus and government commodities.	Standard regional items, local farms, canned meats, produce and holiday ham/turkeys.	Standard goods, frequently emphasizing local farm or community garden produce.	General commercial grocery surplus and shelf-stable staples.	Matzo, gefilte fish, grape juice, and Kosher-certified proteins.	Dates, lentils, chickpeas, basmati rice, and Halal-certified meats.	Flour, lentils (*daal*), rice, ghee.	Rice, lentils, flour, and spices.	Tofu, bulk grains, soy beverages, dried fruits, or mushrooms, and a variety of fresh vegetables.
Holiday adjustments	General winter holiday distributions (Thanksgiving/Christmas).	Major distributions for Thanksgiving, Christmas, and Easter.	Major seasonal food drives aligned with the traditional liturgical calendar.	Large outreach events around Thanksgiving, Christmas, and back-to-school.	Specialized food boxes containing certified **Kosher-for-Passover** items.	Heavy distributions of dates for *Iftar* before Ramadan and meat for Eid al-Adha.	Heavy dates: *Gurpurabs* (Guru birthdays) & *Vaisakhi*.	Major seasonal drives: diwali and major temple festivals.	Increased distributions during Vesak (Buddha's birthday) or ghost festival.
Funding network & NYC legal Year	Government grants, corporate sponsors, and the United Way.	Catholic charities NYC (1812) & US network and parish tithes. Donations.	Episcopal relief & development, parish budgets, and local trusts. Donations	Independent church budgets, congregational donations, and rescue missions.	Jewish Federations and specialized networks like UJCES (1971); Met Council (1975), MASON (1983), & Masbia (2005).	Global and national NGOs like ICNA Relief or Islamic Relief, plus mosque donations.	Local Gurdwaras & Khalsa Aid. United Sikhs (1999)	Local temples, cultural groups, ROCS (2009), Hare Krishna Food for Life (ISKCON, 1966) & SEWA International.	Temple donations, Buddhist foundations (e.g., Tzu Chi Foundation), and monastic communities.

This table reflects the denominations most frequently registered with food pantries in the Bronx. Other unlisted religious groups may offer similar community services.

#### Alimentary quality and the pantry environment

The physical and nutritional environment of food pantries heavily dictates the health outcomes of their participants (47). Historically, reliance on arbitrary private corporate donations or low-quality retail food recovery has led to a highly variable supply chain (45). Traditional packages are frequently deficient in fresh produce, culturally relevant items, and balanced macronutrients, forcing clients to adjust their dietary practices in ways that exacerbate metabolic diseases and fail to guarantee a balanced diet (48–50). While successful interventions (e.g. “Crooked Creek Food Pantry”, “Preventive-Food-Pantry“, “City Harvest”, the “Fresh Shelves”, ”SuperShelf“, ”Healthy Pantry“, “#GiveHealthySystem”) initiatives seek to optimize choice-based models and behavioral economics to nudge clients toward healthier options, resource limitations frequently stall implementation in low-income urban environments (5, 51–55). Urban growth introduces unique environmental constraints including food deserts or apartheid, space limitations, waste management issues, and severe city logistics friction (56).

#### Supply chain fragility and gleaning complexities

The logistics of food pantries are highly unpredictable (45), as their supply chains are vulnerable to global shocks, trade disputes, and public health emergencies that simultaneously increase client demand and reduce food availability (41, 57–59). During such crises, charitable food programs, particularly those led by faith-based organizations, function as essential socioeconomic mechanisms that help participants supplement limited food budgets (60–62). Although decentralized grassroots food recovery and gleaning networks provide vital alternatives for fresh food procurement by connecting local growers directly to pantries (63), they face notable systemic friction. When integrated with food pantries, these initiatives create valuable “third spaces” (64) that redirect agricultural waste into community nutrition, yet frontline operators often struggle to navigate complex food safety and traceability regulations (65). Additionally, decentralized models require precise data optimization, as accurate client profiles enable higher drop density, improving the logistical feasibility and cost-effectiveness of regional delivery. Unlike large food banks with the infrastructure to manage strict compliance protocols and big data software, small faith-based enterprises face acute capacity constraints, such as insufficient cold storage, irregular donation timing, and high volunteer turnover (66). Imposing rigid regulatory burdens without providing structural support directly threatens their institutional survival.

#### Financial instability and management hurdles

Financially, many food pantries exist on the brink of operational insolvency (67). They navigate a continuous cycle of budgetary strain, relying on volatile philanthropic grants, individual congregational tithing, and unpredictable municipal support (68). This financial precarity is worsened by structural funding models where funders are not the direct ”customers” (46). Because philanthropic funders are rarely the direct consumers of the pantries' services, institutions often over-invest in reputation management at the expense of core capability investment, staff professionalization, and technological upgrades. When federal or state funding cuts occur, food banks/pantries are forced to reduce services, leaving patrons without viable alternatives, contributing to a broader trend of nations relying heavily on charitable food infrastructure (69, 70).

Furthermore, managerial capacity within FBFP varies widely (71). Many sites are coordinated by elderly, well-intentioned volunteers or clergy for whom running the pantry is not a full-time occupation (72, 73). Studies indicate that while pantry managers possess highly positive nutrition beliefs, they frequently score poorly on objective nutritional knowledge assessments (62, 74). Moreover, stark demographic disparities often exist between college-educated, non-local pantry directors and the ethnically diverse, low-income client populations they serve, leading to misalignments in cultural relevancy and food preferences. Imposing state compliance standards or sophisticated digital surveillance workflows directly upon frontline volunteers can introduce fatal friction. To address this capacity-digital gap, building a two-tiered system that separates logistics from administration might help faith-based food pantries integrate into broader public health delivery mechanisms.

### Dignity gap

While food pantries reach only a fraction of the food-insecure population (8), those navigating traditional charitable networks frequently encounter structural humiliation, paternalism, and stigma (75, 76). Dominant welfare models reduce individuals to passive beneficiaries, stripping away personal agency while deepening institutional dependency and guilt (63, 75). Furthermore, critics argue that faith-based networks can leverage systemic vulnerability to advance theological capital under the guise of benevolence (77–79). In urban areas like the South Bronx, these psychological barriers are compounded by institutional fear among vulnerable non-citizen families accessing public benefits or formal, non-confessional food pantries (80).

### Why are the FBFPs the best option for the Bronx?

Despite these documented vulnerabilities, FBFP in the South Bronx possess unique structural advantages that make them ideal candidates for transformation into multiservice health centers (38). Rather than building new public health infrastructure from scratch, an evolution toward an integrated health-delivery paradigm leverages existing community infrastructure, expanding the local safety net through collaborative alliances (81, 82). Based on preliminary unpublished data clustered by zip code (10452 and 10451), the South-Bronx contains one of the largest initial groupings of active distribution infrastructures, ideal to begin the detailed mapping and GIS audits (Figure 1).

**Figure 1:**
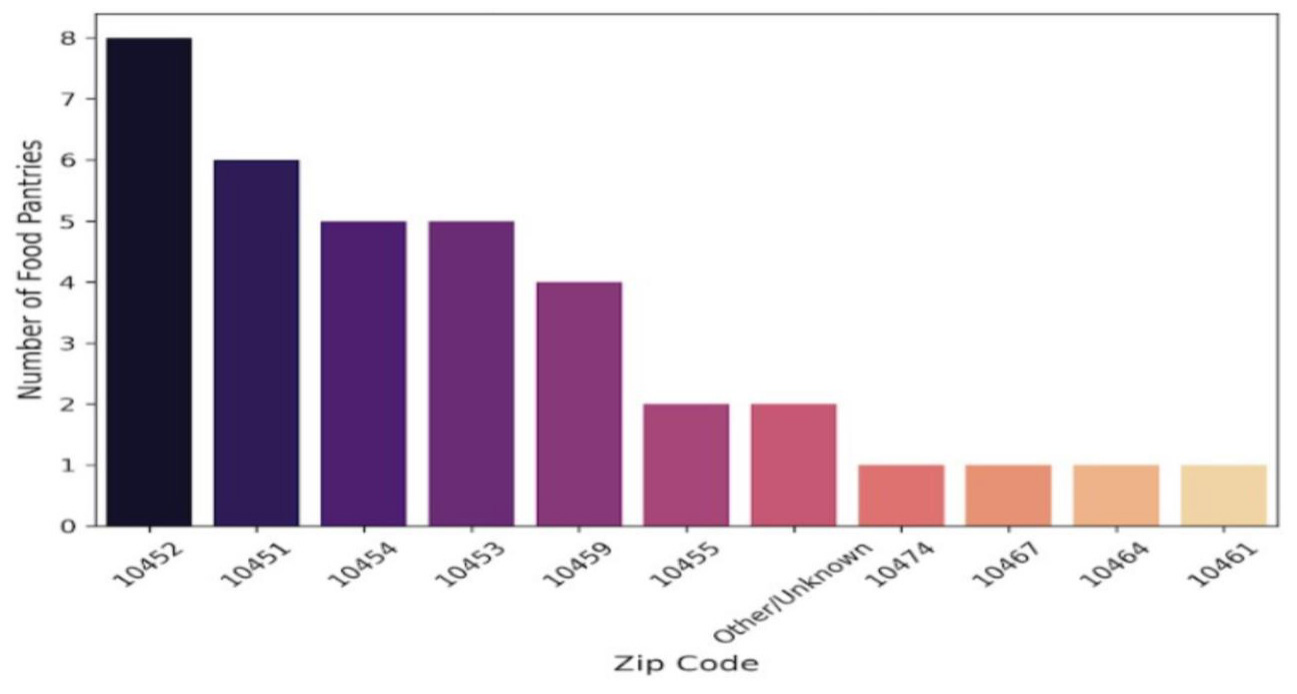
Geographical distribution by zip code (known locations).

Faith-based institutions—such as Catholic, Jewish, and Islamic charities—leverage decades of infrastructure and deep integration with public and private programs. Operating outside strict state surveillance, they offer safe environments for vulnerable clients (80). For example, at a faith-based food pantry (FBFP) simplified mobile interfaces are used by volunteers to record basic, self-reported client data. However, isolating this frontend data from backend cryptographic processing requires robust software (e.g., SAFPAS or Plentiful) to fluidly coordinate client preferences while protecting anonymity (83).

#### Trusted community anchors

The most significant asset of FBFPs is their profound level of community trust (38). In neighborhoods characterized by historical disinvestment, institutional racism, and medical exploitation, marginalized populations are routinely skeptical of traditional healthcare networks and government agencies (26). Religious institutions—whether churches, mosques, synagogues and/or temples—serve as culturally embedded anchors (44, 84). They possess a highly motivated culture of volunteerism and an unmatched ability to cultivate meaningful, face-to-face engagement, often publicizing their services through localized word-of-mouth networks (85).

By embedding health services within a familiar, non-clinical setting, the profound stigma and fear associated with seeking medical, psychological, or social assistance are substantially mitigated (26). Unlike standard clinics, FBFP welcomes anyone with very little paperwork or restrictions. For displaced, immigrant, or highly traumatized populations, faith acts as a complex engine of psychological and social resilience (86–88). When health interventions are aligned with an individual's underlying spiritual belief system, it strengthens their sense of connectedness and control, thereby magnifying the clinical efficacy of the care provided (88, 89).

#### Co-location efficiency

Co-locating healthcare and social services within existing food pantries streamlines service delivery and optimizes non-profit supply chains (90). This strategy eliminates the high overhead and real estate costs of building standalone urban clinics (91) by retrofitting underutilized faith community spaces, such as community halls and classrooms. Furthermore, co-location resolves a primary logistical barrier faced by low-income populations: the challenge of fragmented transit and competing survival priorities (92). When a single geographic visit allows a family to secure fresh, healthy groceries, receive a cardiovascular screening, consult with a licensed social worker, and enroll in social benefits, the artificial barriers dividing nutrition from medicine drop away (81, 82).

#### Prevention, collaboration, and distribution

Transforming pantries into health centers allows for the rapid deployment of holistic preventative care and early-intervention strategies (93). Regular pantry attendees represent a captive audience facing severe chronic health disparities (94). Integrating regular health screenings, vaccine distribution, and targeted health education directly into the pantry flow allows providers to catch metabolic deviations, hypertensive crises, or out-of-control blood glucose levels before they require emergency department care (93, 95). This shift mirrors efforts across similar populations where nutrition security and chronic care tracking are prioritized (96).

Additionally, this transformation facilitates the growth of voluntary, community, social, and faith enterprise (VCSFE) networks (85). Many FBFPs in NYC have reported some type of partnerships with external organizations and or a congregational network. For instance, because sourcing Halal meat can be difficult and expensive, Catholic and Muslim pantries in NY partner up (97). Working collaboratively enables separate, historically isolated organizations to pool resources, co-design culturally competent health services, and build shared outreach platforms that drastically scale up localized public health delivery (98).

### Adapt or collapse: operationalizing a four-phase model

Evolving a FBFP into a vital multiservice health center cannot be achieved through spontaneous altruism; it requires a highly strategic, phased, and co-designed structural framework. I am proposing the following four basic phases.

Phase 1: Mapping, characterization, and GIS analytics. Deploy a multi-method screening approach be tested to map and profile actively operating distribution points within the target geographic area (99). Spatial analysis software can be utilized to plot these locations against local health, demographic, and environmental data to evaluate coverage gaps. Concurrently, digital surveys and communication tools can capture foundational operational profiles, capacity constraints, and service hours.

Phase 2: In-depth stakeholder engagement and receptiveness profiling. Execute concurrent quantitative and qualitative stakeholder evaluations to analyze unique organizational structures and participant needs. This proposed phase involves structured assessments with service providers, operators, and clients to gauge organizational readiness for service integration and to evaluate internal volunteer dynamics. Crucially, since not all FBFP possess the requisite capacity to support the initiative, this step distinguishes adaptable models from those requiring structural-partnership intervention (100). Simultaneously, auditing the operational environment to measure service quality and systemic alignment with participant requirements.

Phase 3: Piloting co-located health clinics (the R3 initiative). Propose delivering robust mobile healthcare in unpredictable grassroots settings through a synchronized, three-tier framework that eliminates permanent staffing costs. First, Rotational Care deploys multi-disciplinary teams on a geolocational circuit to conduct diagnostic screenings at food pantries during peak hours. Second, Real-Time Triage uses automated alphanumeric intake to instantly prioritize patients. Finally, Resource Integration allows on-site case managers to immediately enroll high-risk clients into the system, ensuring seamless continuity of care. University interns will bridge staffing gaps by providing hands-on, on-site training and knowledge sharing

Phase 4: Braided sustainability and coalition building. The final proposed phase suggests establishing formal, long-term partnerships with institutional networks and regional service systems to support continuous delivery. A geolocational schedule can be applied to optimize resource allocation across a network of participating organizations. Also, sustaining grassroots entities with rigid fixed overhead costs requires applying a blended financing paradigm. This approach combines traditional grants with structured institutional reimbursement models to fund health-related interventions and ensure long-term operational viability.

### Culturally embedded and participant-centered co-design

The ultimate success or failure of this multiservice transformation depends on the degree to which programs are generated, co-designed, and supported by and with members of the local faith community, rather than imposed by external academic or medical hierarchies (43, 98). Because organizational capacity varies across FBFPs, implementation requirements are heterogeneous. While nascent or resource-constrained pantries may require intensive, direct collaborative support to execute Phases 1 and 2, established sites with extensive operating experience may navigate these stages with greater operational autonomy. Consequently, to optimize programmatic milestones, community-academic partnerships must adopt a tailored, case-by-case approach. Top-down, technocratic public health interventions routinely fail in marginalized urban settings because they ignore the existing coping capacities, institutional culture, affective dynamics, and practical knowledge of the local populace (89, 101).

Interventions must be deeply embedded in the specific cultural and spiritual texture of the population served (86). For example, health promotion curricula can intentionally draw upon relevant spiritual texts, theological themes of bodily stewardship, or communal traditions to encourage positive behavioral modifications and healthy habits (88, 102). Such frameworks must also account for cultural dynamics within dining and hospitality sectors to fully understand underlying faith behaviors around nutrition (103). Furthermore, recognizing the highly gendered nature of community care is vital. Empirically, women frequently assume disproportionately higher relational, domestic, and volunteer responsibilities within both religious institutions and household management (104). Program co-design must intentionally support, respect, and avoid exploiting these female leadership structures, providing them with advanced training, technological tools, and formal support (74).

By utilizing a community-based participatory research (CBPR) lens, the traditional, stigmatizing hierarchy of the “charitable provider” and the “passive recipient” is dismantled (26). Clients must be redefined as active participants and co-producers of knowledge (101). When a faith-based center honors a participant's individual life trajectory, cultural identity, and culinary wisdom, it fosters individual agency, a sense of belonging, and a pathway toward active participation and sustainability (105). This emotional and cognitive alignment transforms the act of receiving aid from an experience of humiliation into an engine of individual empowerment, community resilience, and social justice (42, 75).

#### Securing the trusted firewall

Reflecting on the operational vulnerabilities inherent in volunteer-reliant, FBFP, this framework proposes a structural evolution: a centralized community-university partnership designed to absorb the burdens of administration, healthcare compliance, and data governance. Rather than forcing grassroots religious institutions to adapt to rigid corporate models, this multi-sectoral alliance propose to integrate university student internships, community benefit alignments, and federally qualified health center networks to build a protective data firewall between healthcare norms, the clinical systems and the localized delivery logistics. By tokenizing patient intake profiles into de-identified spatial nodes, the university logistics hub can optimize high-density regional circuits using only alphanumeric identifiers.

To protect highly vulnerable, non-citizen, and traumatized populations from neighborhood surveillance and immigration data-tracking, the community-university alliance operates a strict data firewall utilizing Zero-Knowledge Proof (ZKP) protocols.

Backend tokenization: This raw data is instantly encrypted and sent to the university-hospital logistics hub, which converts the profile into a de-identified alphanumeric spatial node.

Cryptographic validation: Through ZKP architecture, the university backend generates a verifiable cryptographic proof confirming that an eligible individual received a specific health intervention (e.g. diet-tailored meal box).

In other words, deploying ZKP protocols enables the intermediary academic institution to manage administrative claims and fiscal reimbursements without breaching the protective data firewall established around FBFPs. Consequently, the community-university alliance can supply state health agencies and Managed Care Organizations (MCOs) with auditable, verified compliance metrics while preserving the total anonymity of participants' identities, precise locations, and other sensitive demographic data.

Crucially, this structural integration preserves religious liberty and complies with non-establishment mandates by leveraging multi-sector partnerships to promote holistic wellbeing for populations in need. Existing New York State statutory and regulatory frameworks facilitate access to healthcare services for uninsured, indigent, and undocumented or non-citizen residents. Under this blended financing paradigm within the Bronx context, while the community-university partnership manages administration, healthcare compliance, and data governance, philanthropic capital provides upfront bridge funding to de-risk initial capital outlays and basic technical infrastructure. Concurrently, public healthcare mechanisms sustain long-term operations: under frameworks such as New York's Section 1115 health-related social needs (HRSN) waiver, Medicaid Managed Care Organizations (MCOs) route service reimbursements through designated social care networks (SCNs) or community care hubs (CCHs), using validated digital tokens to trigger automated operational payouts. This dual-stream model establishes fiscal sustainability, leverages privacy-preserving technologies, and formalizes a volunteer-to-Community Health Worker pipeline that cultivates local leadership and strengthens community agency.

#### Beyond intuition

To move this conceptual framework into the domain of reproducible public health science, the academic community must prioritize three distinct empirical validation tracks:

Track 1 (implementation): evaluate how FBFP organizational readiness, nutritional and public-health facilitation capabilities by validated tools (e.g. NEFPAT plus).

Track 2 (health economics): build cost-benefit models to quantify how FBFP holistic-preventative screenings reduce avoidable, Medicaid-funded emergency room admissions (e.g. hypertensive identification and food-choice).

Track 3 (digital privacy): execute cluster-randomized trials comparing client utilization rates between state-surveilled tracking software and privacy-shielded, ZKP-enabled app interfaces.

## Conclusion

Reconfiguring faith-based food networks into multiservice health centers offers a scalable framework to mitigate entrenched public health disparities in underserved regions such as the Bronx, NY. By leveraging the unique cultural alignment and trust of local religious entities, this model actively counters paternalistic critiques, transitioning grassroots networks from transient relief providers into enduring engines of health equity and community empowerment. While a standardized, fully documented blueprint has yet to be established, realizing the transformative potential of this paradigm ultimately depends on robust cross-sector partnerships, sustainable Medicaid financing, and a shared commitment among stakeholders to prioritize the common good over institutional self-interest.

## Data Availability

The raw data supporting the conclusions of this article will be made available by the authors, without undue reservation.
